# Roles of IL‐2 in bridging adaptive and innate immunity, and as a tool for cellular immunotherapy

**DOI:** 10.1002/JLB.5MIR0420-055R

**Published:** 2020-06-01

**Authors:** Kamila Bendickova, Jan Fric

**Affiliations:** ^1^ International Clinical Research Center St. Anne's University Hospital Brno Brno Czech Republic; ^2^ Institute of Hematology and Blood Transfusion Prague Czech Republic

**Keywords:** myeloid cells, monocytes, cyclosporine A, Tacrolimus, calcineurin inhibitors

## Abstract

IL‐2 was initially characterized as a T cell growth factor in the 1970s, and has been studied intensively ever since. Decades of research have revealed multiple and diverse roles for this potent cytokine, indicating a unique linking role between adaptive and innate arms of the immune system. Here, we review the literature showing that IL‐2 is expressed in a plethora of cell types across the immune system, where it has indispensable functions in orchestrating cellular interactions and shaping the nature and magnitude of immune responses. Emerging from the basic research that has revealed the molecular mechanisms and the complexity of the biologic actions of IL‐2, several immunotherapeutic approaches have now focused on manipulating the levels of this cytokine in patients. These strategies range from inhibition of IL‐2 to achieve immunosuppression, to the application of IL‐2 as a vaccine adjuvant and in cancer therapies. This review will systematically summarize the major findings in the field and identify key areas requiring further research in order to realize the potential of IL‐2 in the treatment of human diseases.

## Graphical abstract

Review on IL‐2 in adaptive and innate immunity and its potential applications in immune therapies.

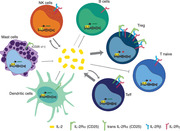

**Abbreviations**
AML: acute myeloid leukemia
CAR: chimeric antigen receptor
CN: calcineurin
CsA: cyclosporine A
DC: dendritic cell
GVHD: graft‐versus‐host disease
TIL: tumor‐infiltrating lymphocytes
Treg: T regulatory cells

## 1 INTRODUCTION

IL‐2 was originally identified in the 1970's as the first T cell growth factor.^1^, ^2^ As a result of this key property, it has been intensively researched ever since: thousands of papers now describe the details of IL‐2's molecular and cellular biology (reviewed in Refs. 3,4 and others). What has emerged from these studies is a complex picture that extends well beyond the limited scope of a prototypical T cell growth factor; in fact, IL‐2 occupies a central position in all immune responses and during homeostasis, being produced by, and acting upon, a plethora of cell types, with its effects determined by source, target, dose, and context.

Our understanding of IL‐2's roles is underpinned by early studies that elucidated its molecular mechanism of expression in T cells, which led to the pivotal discovery of the NFAT family of transcription factors.^5^, ^6^ These studies showed that T cell receptor stimulation results in the opening of calcium channels in the cell membrane, and so increases levels of intracellular calcium, leading to activation of the calcium‐ and calmodulin‐dependent serine/threonine protein phosphatase, calcineurin (CN). Activated CN then dephosphorylates NFAT in the cytoplasm, causing a change in its conformation that exposes a nuclear translocation sequence, leading to initiation of transcription of target genes, including of *il2*.^7^, ^8^ What we now know is that this process does not occur only in T cells, but also in cells of the innate immune system, such as dendritic cells (DC), monocytes and NK cells.^9^

NFAT family members are widely expressed across the immune system, but it is NFAT1 and NFAT2 that are required for IL‐2 production.^10^, ^11^ Despite this dependence, NFATs alone are unable to initiate gene expression, and require cooperation with other transcription factors including NF‐kB,^12^ AP‐1,^13^ or T‐bet^12^ to promote IL‐2 expression in different cell types. In contrast, in T regulatory cells (Treg), the binding of NFAT together with the FoxP3 transcription factor represses IL‐2 expression,^14^, ^15^ explaining the dependence of Treg on IL‐2 from other cellular sources. Altogether these molecular interactions aim to assure balanced expression of IL‐2 in innate and adaptive immune cells, which is essential for orchestrating an optimal immune response.

One reason that the discovery of NFAT as a molecular regulator of IL‐2 signaling was so important is that it led to a means to target IL‐2 expression and achieve clinical immunosuppression. It was first shown that inhibition of the phosphatase activity of CN using the fungal isolate cyclosporine A (CsA) or the synthetic inhibitor FK506 resulted in impaired NFAT signaling,^8^, ^16^ leading to reduced expression of IL‐2, and an efficient immunosuppressive effect.^16^ These immunosuppressive therapeutic approaches have since revolutionized the field of organ transplantation, reducing the rejection of solid grafts,^17^ and limiting graft‐versus‐host disease (GVHD) in hematopoietic stem cell transplant recipients.^18^, ^19^ The success of CN inhibitors in the transplantation setting led to trials and eventual widespread use of CsA and FK506 in other pathologies, including psoriasis, eczema, rheumatoid arthritis, and Crohn's disease.^20^ However, simultaneously, the recognition of the importance of IL‐2 for maintaining immune‐suppressive Treg has led to the somewhat counterintuitive strategy of treating some autoimmune conditions with low doses of exogenous IL‐2 summarized in Table 1. Together, these opposing strategies demonstrate the complexity of IL‐2's roles across the immune system, and the ongoing challenges of understanding this enigmatic cytokine.

**TABLE 1:** Overview of different approaches to the therapeutic use of IL‐2

Group of disorders	Disease	References	IL‐2 dose	Note	Major findings
Autoimmune diseases	Systemic lupus erythematosus	^21^, ^22^, ^23^, ^24^, ^25^	Low		IL‐2 corrected Treg defects, promoted Treg /NK cell expansion → restoration of immune homeostasis
	HCV‐induced vasculitis	^26^	Low		IL‐2 led to Treg recovery without adverse effects
	Type 1 diabetes	^27^, ^28^, ^29^, ^30^	Low		Determination of optimal doses of aldesleukin needed to expand Tregs. Partial desensitization of Treg to IL‐2 on day 3 after treatment → improvement of dosing regimens for future trials. Selective Tregs responses to low IL‐2 through IL‐2‐dependent transcriptional amplification mechanism.
	Alopecia areata	^31^	Low		IL‐2 led to increased Treg count. No adverse event was reported.
	Rheumatoid arthritis	^25^	Low		IL‐2 induced Treg expansion and activation without effector T cell activation.
	Crohn's disease, ulcerative colitis	^25^	Low		IL‐2 induced Treg expansion and activation without effector T cell activation.
Transplantations	Graft‐versus‐host disease	^32^, ^33^, ^34^, ^35^	Low		IL‐2 administration was associated with preferential, sustained expansion of functional Tregs (while maintaining the immune response to infections) resulting in reduced chronic GVHD. IL‐2 restores homeostasis of CD4^+^ T cell subsets through selective increase of Stat5 phosphorylation in Tregs and a decrease of phosphorylated Stat5 in conventional CD4^+^ T cells.
Inflammatory condition	Chronic kidney disease (CKD)	^36^	Low		Treg count is lower in CKD patients. IL‐2 selectively expanded CD4^+^CD25^hi^ and CD4^+^CD25^+^FoxP3^+^ Tregs and up‐regulated the expression of FoxP3 mRNA.
	Cardiovascular diseases (preclinical model)	^37^	Low	IL‐2/anti‐IL‐2 complex (IL‐2C)	Tregs can suppress immunologic damage in myocardial ischemia/reperfusion injury (MIRI). IL‐2C led to Treg expansion resulting in attenuated MIRI and improved myocardial recovery (mouse model).
Infection	Tuberculosis (TB)	^38^, ^39^	High		IL‐2 immunotherapy appears to promote the proliferation and conversion of CD4^+^ (Th1) and NK cells and decrease Th17 and Treg populations. The improved sputum culture and smear conversion of TB patients were reported.
	Persistent viral infections	^40^		IL‐2C	IL‐2C improved IL‐2 signaling and enhanced the quality of the CD8 T cell response via up‐regulation in granzyme B production increasing cytotoxicity and higher numbers of virus‐specific CD8 T cells (mouse model).
	HIV	^41^, ^42^		Intermittent administration of IL‐2	IL‐2 induced sustained increase of CD4^+^ T cells.
Cancer	Melanoma	^43^, ^44^, ^45^, ^46^	High		IL‐2 therapy displayed durable response and antitumor activity in some patient with metastatic melanoma. Selective inhibition of IL‐2‐mediated enhancement of T may be beneficial for IL‐2 therapy.
	Renal cancer	^45^, ^46^	High		IL‐2 therapy displayed durable response in subset of patients. Selective inhibition of IL‐2‐mediated enhancement of T may be beneficial for IL‐2 therapy.
	Acute myeloid leukemia	^47^, ^48^, ^49^, ^50^, ^51^	High, low, IL‐2 diphtheria toxin (IL2DT)		IL‐2 used to expand the number of circulating NK cells before high‐dose chemotherapy and autologous hematopoietic cell transplantation. Depletion Tregs with IL2DT improved efficacy of haploidentical NK cell therapy. HD IL‐2 therapy was related with adverse reactions.
	Lymphoma	^52^			IL‐2‐activated cells generate PBMCs with enhanced cytotoxicity against NK‐resistant targets, and increase cytokine levels.
	Multiple myeloma	^53^			IL‐2 and phosphostim stimulate in vitro expansion of γδ T cells which are efficient in killing of human myeloma cells.

**FIGURE 1:**
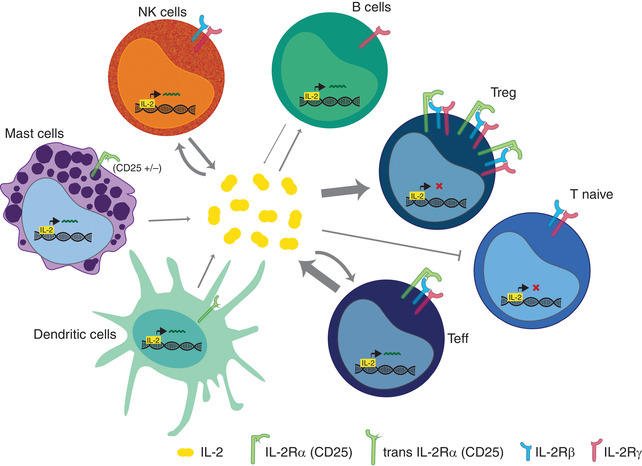
**IL‐2 as a bridge between adaptive and innate immunity**. Both adaptive and innate immune cell subsets have been identified as a source of IL‐2. Here, we summarized the main IL‐2 producers as well as the capacity of these cells to bind IL‐2 via 1 of the IL‐2 receptors: low affinity IL‐2Rα (CD25) dimeric intermediate affinity IL2‐R consisting of IL‐2Rβ (CD122) and the common γ ‐chain (CD132) and high affinity IL‐2R—heterotrimer of subunits α, β, and γ‐chain. Activated T cell represents main producer of IL‐2, although IL‐2‐driven regulation of B cell fate has been shown and to certain extent B cell are able to produce IL‐2 as well. Naïve T cells are not responsive to IL‐2 and they lack CD25. During early stage of immune response, after antigen activation, the expression of CD25 is induced leading to IL‐2 expression, T cell proliferation, and differentiation. Unlike other T cell subsets, Treg cells constitutively express high levels of CD25, with intermediate levels of CD122 and γ chain and are dependent on exogenous source of IL‐2. Within innate immune cells IL‐2 expression was proved in NK cells, activated DC and mast cells. IL‐2 is necessary for many NK cell functions. Although NK cells secrete IL‐2, they are likely dependent on T cell derived IL‐2, whereas intermediate affinity receptor helps to bind IL‐15. Some subsets of mast cells express IL‐2Rα although likely independently of its IL‐2‐related functions.^58^ DC are able to trans present IL‐2 through CD25 and thus mediate T‐cell activation

Alongside progress in understanding NFAT‐mediated signaling leading to IL‐2 production, a further layer of complexity has more recently become apparent from studies of IL‐2's downstream signaling in target cells. Complex molecular mechanisms control cellular sensitivity to IL‐2. One mechanism is through variable subunit composition of the heterotrimeric IL‐2 receptor (IL‐2R), whose affinity for the cytokine is determined by the combinations of the IL‐2Rα (CD25), IL‐2Rβ (CD122), and the common gamma or γ_c_ (CD132) chains.^3^, ^54^ The “low affinity” IL‐2R is formed only by IL‐2Rα, whereas the “intermediate affinity IL‐2R” consists of IL‐2Rβ and γ_c_, and the “high affinity IL‐2R” is comprised of 1 of each of the 3 chains.^55^ The expression of different versions of the IL‐2R on responder cells (also shown in Fig. 1) has a profound impact on the effects of IL‐2 on those cells (reviewed Ref. 56). Furthermore, the IL‐2Rβ and γ_c_ chains are also able to bind IL‐15 and IL‐21,^3^, ^57^ which are involved in the regulation of immunologic processes through overlapping actions or by competing with each other, and with IL‐2, for receptor engagement.

Therefore, although the first role of IL‐2 in T cells was described 50 years ago, advances in our understanding of the regulation of its production and signaling, and how it might be used effectively and precisely to modulate patients’ immune systems, are still very much a topic of ongoing research. This review will examine recent findings in these areas, with particular emphasis on IL‐2's emerging roles in the crosstalk between innate and adaptive immune cells, and its current and future uses in clinical immunotherapy.

## 2 ROLES OF IL‐2 IN ADAPTIVE AND INNATE IMMUNITY

### 2.1 Roles of IL‐2 in orchestrating adaptive immunity

IL‐2 is a major modulator of the development, homeostasis and functions of various T cell subsets, and therefore has key role in orchestrating the balance of adaptive immune responsiveness. It has long been known that in the thymus IL‐2 fuels the initial proliferation of naïve T cells^59^ and is essential for maturation of Treg.^60^ At the same time, IL‐2 is also responsible for the expansion and cytotoxicity of effector T cells.^56^ What remains debated is which cell types in the thymus are the key cellular sources of IL‐2 for the different lymphocyte subtypes. For example, in the case of murine Treg, while 1 study showed that DC‐derived IL‐2 was important for their development in an ex vivo thymic slice model,^61^ another group using *IL‐15* ^−/−^ mice with *il‐2* also deleted in T cells, B cells and DC, reported that only T cell‐derived IL‐2, and not IL‐2 from B cells or DC, was essential for Treg development in the thymus in vivo.^62^

In the periphery, IL‐2 is a master regulator of T cell biology. Effector T cells are the main producers of IL‐2 that they use for autocrine stimulation of their own proliferation, cytotoxicity, and the downstream development of memory T cells.^63^ T cell homeostasis also relies on paracrine IL‐2 signaling.^64^ Interestingly, studies on human DC have revealed their ability to capture and present either DC‐ or T‐cell produced IL‐2 at the immunologic synapse in order to stimulate antigen‐specific T cell proliferation.^65^ These findings highlight a novel mechanism by which even extremely small amounts of IL‐2 can be critical for the initiation of immune responses by acting, quite literally, as a molecular bridge/connection between the effector cells of the innate and adaptive arms of immunity.

Although the roles of IL‐2 in stimulating immune responses are well known, early studies in mice lacking IL‐2 or its α or β receptor chains also uncovered the role of IL‐2 in preventing autoimmunity,^66^, ^67^, ^68^ which we now know relates to the dependence of Treg on this cytokine for their development and maintenance.^64^ Similar to effector T cells in the periphery, studies in the mesenteric lymph nodes have revealed the importance of both T cell‐ and DC‐ derived IL‐2 in Treg homeostasis. For example, in the gut mucosa, tolerance is largely maintained by Treg,^69^, ^70^, ^71^ with IL‐2 playing a key role via a range of mechanisms: mucosal Treg are maintained by the IL‐2 from naive CD4^+^ T cells^72^; whereas in parallel, IL‐2‐driven Treg development inhibits the differentiation of naïve CD4^+^ T cells into Th17 cells,^73^ though the cellular source of this IL‐2 is unknown. It is an open question whether the same cellular sources are important for Treg maintenance across all lymphoid tissues, or whether the dominant cellular source of this cytokine varies by microenvironment. Alongside its importance for Treg functions, recent data suggest that mucosal‐associated invariant T cells, which are innate T cells, necessary for gut immune system regulation, are also dependent on IL‐2.^74^ Taken together these studies show how IL‐2 produced by innate immune DC and adaptive immune T cells, in the gut in particular, have distinct but complementary roles in managing the immune environment in the periphery. Whether IL‐2's role was essential or was overlapping/redundant with that of other cytokines sharing the same beta and gamma receptor chain, such as IL‐15, was for a long time controversial; nevertheless, experiments in mice with an IL‐15^−/−^ background have now distinguished specific functions of IL‐15 in the maintenance of CD8^+^ memory T cells, whereas IL‐2 is indispensable for the maintenance of Treg.^69^, ^75^

Overall, it is now clear that IL‐2 orchestrates T cell homeostasis through several different mechanisms ranging from paracrine signaling^64^ to cross‐presentation of T cell produced IL‐2 by CD25‐expressing DCs during the T–DCs interaction.^65^ In summary IL‐2 from both innate and adaptive immune cell sources plays the key role in T cell activation during the primary immune response and throughout reactivation of memory T cells; furthermore the other important role of IL‐2 is to establish negative regulatory feedback loop around the T cell response by driving the expansion of Treg populations.^3^ The plasticity of T cell subsets’ capacity to produce or sense IL‐2 creates a complex regulatory environment controlling the process of adaptive immune responses on different levels. IL‐2 is indispensable for the regulation of both immune activation and immunosuppressive responses to foreign or self‐antigens, and is the key to homeostatic maintenance of T cell populations.

### 2.2 Roles of IL‐2 in orchestrating innate immunity

#### 2.2.1 Myeloid cells and IL‐2

Although IL‐2 was long‐considered purely a T cell cytokine, there is clear evidence that functional calcium‐NFAT signaling also occurs within some myeloid cell subsets, as reviewed.^20^, ^76^, ^77^ Activation of the CN‐NFAT pathway was first described in DC in response to whole bacteria or LPS,^78^, ^79^ and since, also in response to stimulation with the fungal components zymosan,^80^ or curdlan.^81^ These findings led others to investigate the activation of the CN‐NFAT pathway in macrophages, which was found to be stimulated upon phagocytosis of fungal conidia,^82^, ^83^ and in human macrophages by exposure to *Aspergillus fumigatus*.^84^

Intensive research followed these initial findings, aiming to establish the molecular mechanisms of NFAT activation in myeloid cells. Together, they revealed multiple pathways leading to CN‐NFAT signaling: in murine macrophages and DC, Dectin‐1 ligation by yeast or zymosan particles resulted in NFAT activation^85^; whereas murine DC exposed to LPS or whole bacteria showed CN‐NFAT activation followed by IL‐2 expression that relied on TLR4 ligation.^78^, ^79^ Later studies refined this work by showing that CD14 was capable of mediating bacterial ligand‐induced CN‐NFAT activation alone in these cells.^86^ Furthermore, the interaction of TLR‐9 and Dectin‐1 with β‐glucans within the fungal cell wall leads to activation of Burtons tyrosine kinase (BTK)‐CN‐NFAT in murine macrophages during experimental pulmonary aspergillosis.^82^ Last, paralleling aspects of T cell biology, the ligation of Dectin‐1 and other C‐type lectin receptors on murine macrophages initiates the activation of the ITAM, resulting in NFAT activation through Syk phosphorylation.^85^

Perhaps due to the pervading dogma of IL‐2 as a T cell cytokine, few studies that reported CN‐NFAT activation in myeloid cells initially looked for IL‐2 expression. Therefore, the ability of NFAT to initiate IL‐2 transcription in myeloid cells, and the possible biologic relevance of this, remained an open question for many years. Granucci et al.^78^, ^79^ first identified the expression of IL‐2 in murine DC, but it was more than a decade before Yu et al.^87^ successfully used a human DC cell line to perform genome‐wide mapping and found the target sites of NFAT1 binding, confirming that NFAT1 was able to modulate expression of IL‐2 in human DC.

At around the same time, evidence began to build for an important biologic role of innate immune cell‐derived IL‐2 in modulating systemic adaptive immune responses. Mice lacking IL‐2 expression in CD11c^+^ cells were found to express higher levels of IL‐17 in their lungs, resulting in increased susceptibility to infection with *A. fumigatus* as a result of a pathologic Th17 response.^88^ Parallel clinical observations were made in human patients, where there is some evidence that treatment with CN inhibitors increases patients’ susceptibility to fungal infections, but the overall picture remains somewhat unclear.^20^, ^89^ A similar approach has also been used to investigate the role of myeloid cell‐derived IL‐2 in gut homeostasis. Mencarelli et al.^90^ compared mice lacking either CN or IL‐2 in either CD11c^+^ or LysM^+^ cells, and showed that impairment of CN or of IL‐2 in myeloid cells leads to spontaneous intestinal inflammation and increased susceptibility to experimental colitis. Interestingly, they also found that distinct DC subsets in the gut produce different levels of IL‐2, with CD103‐expressing DC the most, although the biologic significance of this difference is not yet known. Interestingly, this study also confirmed earlier findings by Han et al.^91^ revealing a CN‐NFAT signaling‐independent mechanism of IL‐2 production in murine DC, instead utilizing the TLR‐TRAF6‐NFκB cascade. In the Han study, mice lacking TRAF6 specifically in DC exhibited spontaneous enteritis that was associated with unrestrained Th2 responses and decreased Treg numbers; importantly, DC in these mice expressed significantly lower levels of IL‐2, and the aberrant immunophenotype could be rescued by the addition of exogenous IL‐2,^91^ implicating DC‐derived IL‐2 in the maintenance of adaptive immune tolerance in the murine gut. Intriguingly, the same study also identified the microbiota as an important component of the TRAF6‐deficient DC immunophenotype, but the mechanisms underlying this phenomenon and the relationship between intestinal microbiota, DC‐derived IL‐2 and T cell homeostasis in the gut were elusive. Recent findings may, however, provide a further piece of the jigsaw: Zhou et al.^92^ found that innate lymphoid cells are also important producers of IL‐2 in the murine intestine, dependent on macrophages being stimulated by the microbiota to produce IL‐1β. Importantly, this study also showed that innate lymphoid cells from patients with Crohn's disease produced significantly less IL‐2, which was associated with lower frequencies of Treg in their intestine.

Taken together, there is now clear evidence for an important role of innate cell‐derived IL‐2 in mediating adaptive immune homeostasis and responses to microorganisms (whether pathogenic or commensal) in the lung and gut. Future studies are needed to resolve which cell types are important in different locations/situations within these tissues, and also in different tissues. Pressing research questions remain particularly in the area of tolerance in the gut, driven by innate cell‐derived IL‐2 production, and linked to interactions with the microbiota. Given the observations in human Crohn's patients made by Han et al., further studies in this area could be highly productive in understanding immune‐related gut pathologies and in developing improved immune‐targeted treatments.

#### 2.2.2 NK cells and IL‐2

Immune surveillance provided by NK cells is a key mechanism to eliminate infected or cancerous cells. NK cell expansion, maturation, activity, and cytotoxicity are strongly dependent on levels of IL‐2.^54^, ^93^, ^94^ Although NK cells are able to express their own IL‐2 upon activation of the CN‐NFAT pathway,^95^ other reports have described their dependence on IL‐2 produced from T cells,^96^ and, more recently, on DC‐derived IL‐2 for activation of IFN‐γ production.^97^, ^98^

IL‐2 also sits at the crossroads of innate NK cell regulation and the regulation of Treg from the adaptive arm of the immune system. In NK cells, the absence of various chains of the IL‐2 receptor, or the lack of IL‐2, results in impaired NK cell homeostasis.^56^ In particular, CD127^+^ immature NK cells expand in an IL‐2‐dependent manner, which is strongly inhibited by presence of Treg due to competition for IL‐2; accordingly, depletion of Treg results in expansion of NK cell numbers.^99^ Mechanistically, CD127^+^ NK cells are thought to compete for IL‐2 binding with Treg via expression of the high affinity IL‐2Rα, whereas the IL‐2Rγ_c_ chain, which recognizes IL‐2, IL‐15, and IL‐21, mediates the multiple facets of NK cell activation, maturation, and proliferation that are modulated by these closely‐related cytokines.^100^, ^101^

## 3 IL‐2 IN IMMUNOTHERAPY

The prominent role of IL‐2 in T cell stimulation led to it being the first human cytokine employed therapeutically. Almost 4 decades ago, IL‐2 was used with some success to treat cancer,^102^ and today, researchers continue to dissect its importance in this disease, with a recent study showing that single nucleotide polymorphisms in the IL‐2 gene are associated with colorectal cancer prognosis.^103^ However, the range of conditions that IL‐2 is used to help treat stretches beyond cancer to autoimmunity and chronic infections. Therapeutic approaches involving IL‐2 can be broadly divided into those using the cytokine directly in vivo either alone or together with adoptive cell transfer, and those employing IL‐2 for the pretreatment or expansion of therapeutic cells in vitro.

### 3.1 Direct uses of IL‐2 in vivo or in combination with adoptive cell transfer protocols

**FIGURE 2:**
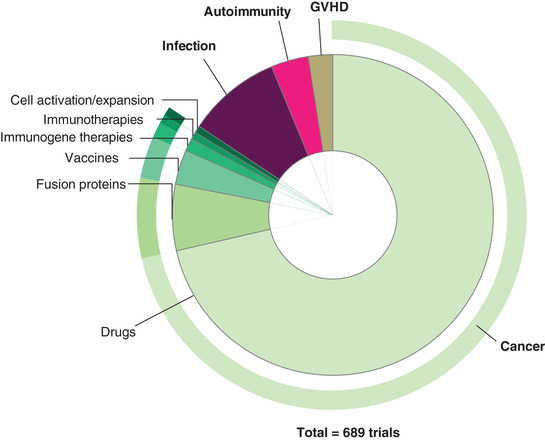
Overview of recent clinical studies using IL‐2 alone or in combination with other drugs in the therapy against cancer, autoimmune disorders, graft‐versus‐host disease, or infection

Intense research and numerous clinical trials culminated, in 1992, in the approval of IL‐2 infusion as the first licensed immunotherapy for the treatment of renal cancer.^102^ Since then many clinical trials testing IL‐2's ability, either alone or in combination with other therapeutic approaches, to treat a range of conditions have been conducted or are ongoing (Fig. 2). Although high‐dose IL‐2 remains a good treatment option for a subgroup of metastatic renal cancer and melanoma patients, its more widespread use has been limited by its toxicity, relatively short half‐life, and variability in patient responses.^104^ Despite this, therapy related mortality remains very low, and IL‐2 treatment can offer significant improvements to survival, particularly in patients with metastatic melanoma.^43^

Although high‐dose IL‐2 therapy has mostly been used to target late‐stage cancers, low‐dose infusion therapies were developed to promote Treg expansion and thereby treat autoimmunity and disorders of inflammation. Following promising data from experimental models, the first clinical studies in 2011 confirmed that low‐doses of IL‐2 were effective in treating GVHD and hepatitis virus C‐induced vasculitis via their proliferative effects on Treg.^26^, ^32^

This proven potential leads to ongoing research into ways to optimize IL‐2 therapy, by manipulating, for example, dosage or route of administration, or via the administration of engineered IL‐2 derivatives (see trails listed in Table 1 and strategies illustrated in Fig. 2). An important development has been the generation of protocols for the production of clinical grade recombinant fusion proteins. This allowed the first progress towards extending IL‐2's half‐life, by using an engineered IL‐2 analog with improved binding and activator function.^105^ The same technology also permitted the investigation of strategies targeting IL‐2 to specific cells at the site of action. For example, a fusion molecule of a fragment of diphtheria toxin conjugated to IL‐2 (Ontak^®^) or its improved equivalent E7777 has been investigated for its effectiveness in Treg depletion in clinical trials.^106^, ^107^ In this case, when the fusion protein is bound and internalized by cells expressing IL2‐Rα, the diphtheria toxin is released from acidic vesicles into the cytoplasm where it inhibits protein synthesis, leading to subsequent cell death. Nevertheless, the safety, efficacy as well as exact effect of this agent on immune cells remain to be elucidated.

Other novel approaches include administration of IL‐2 in immune complexes. These have been used to activate the immune system during chronic viral infections, such as HIV or herpes, where the IL‐2 therapy resulted in increased numbers of Th cells.^40^, ^41^, ^42^ Recent reports also describe the successful combination of IL‐2 treatment with cell cycle checkpoint inhibitors, which was able to overcome previous resistance to these drugs in patients with advanced melanoma,^108^ paving the way for further studies on the adjuvant use of IL‐2 to improve responses to existing chemotherapeutic agents.

In summary, IL‐2 continues to be used and explored as part of the therapeutic protocol for a large number of diseases (Table 1); however, its success has until now been limited due to a short in vivo half‐life, its toxicity, and the cytokine's ability to amplify Treg. More recent studies have focused on how to overcome these drawbacks, and have identified the use of engineered forms of IL‐2,^109^, ^110^ and its combination with traditional chemotherapeutic drugs^108^ as highly promising strategies. A pressing area in need of further work is how to understand which patients will benefit most from IL‐2 immunotherapies, and the mechanisms underlying variable responses to these treatments.

### 3.2 Uses of IL‐2 in cellular immunotherapy expansion protocols

Together with the success of IL‐2 direct infusion therapies, this cytokine is also an indispensable tool for the in vitro expansion and activation of T cells including chimeric antigen receptor (CAR) T cells, γδ T cells or NK cells prior to adoptive transfer. The first clinical studies in this area, over 3 decades ago, used IL‐2‐expanded autologous tumor‐infiltrating lymphocytes (TIL) to treat metastatic melanoma.^111^ However, the application of this approach to other cancers was limited by low expansion of TIL from some patients, the lack of available TIL from other tumor types, and tumor evasive mechanisms. To harness the power of IL‐2 while overcoming the issues associated with TIL transfers, researchers turned to NK cells.

NK cells can exert powerful antitumor effects, which led to interest in their use for cancer immunotherapy.^112^, ^113^ Initial studies used the patients’ own purified NK cells that had been cultured in vitro with IL‐2, and transferred back into the patient following lymphodepletion. In patients with advanced renal cancer or melanoma, the cultured NK cells were able to lyse tumor cells in vitro but did not induce clinical responses in vivo.^114^ Investigations in mice suggested that combining IL‐2 with the closely related cytokine IL‐15, and IL‐18, could generate NK cells with antisolid tumor actions that were retained in vivo.^115^ Since then, improvements in expansion protocols coupled with the elimination of normal lymphocytes through nonmyeloablative conditioning has increased the success of clinical studies in this area (reviewed in Ref. 102).

Related work has focused on the application of IL‐2 during haplo‐identical NK cell infusion treatment for hematologic malignancy. Initial studies reported complete hematologic remission in 30% of poor‐prognosis acute myeloid leukemia (AML) patients treated with subcutaneous IL‐2 after NK cell infusion^50^; however, the success of this strategy was limited by the rapid expansion of Treg cells following IL‐2 administration.^51^, ^116^ To overcome this, following promising data in mice,^92^, ^117^ an engineered IL‐2 diptheria toxin construct was used to deplete Treg in AML patients prior to NK cell infusion; this resulted in increased donor NK cell expansion post‐transplant and significant improvements in remission and survival rates.^51^

Alternative strategies have looked at the use of an NK cell line, NK‐92 genetically engineered to express own IL‐2, in cellular immunotherapies for pathologies that are difficult to target with CAR T cells, such as AML.^118^, ^119^ Preclinical testing resulted in promising findings as NK‐92 improved survival in an AML allograft model.^120^ This approach is paving the way for the use of genetically‐modified NK cells in the clinic.

As well as NK cells, γδT cells also have the potential to express strong antitumor activities.^121^, ^122^ When γδT cells are expanded in vitro in the presence of IL‐2 and a synthetic agonist,^53^ or IL‐15 ^123^, ^124^, ^125^ they exhibit powerful killing of human cancer cells and cell lines. These promising data led to trials in human cancer patients. When daily IL‐2 treatment was used as part of an autologous γδT cells transfer protocol in a small group of people with advanced hematologic malignancy, 3 out of 4 achieved complete remissions lasting between 2 and 8 months.^126^

Here, we have summarized the main strategies used to expand cells for adoptive transfer therapies. Of note, the majority of the reported studies have shown success using these immunotherapies in patients with late stage and/or refractory disease, some of whom had already been treated unsuccessfully with IL‐2 alone or by hematopoietic stem cell transplant. These therapies are likely to be even more successful in patients with earlier stage disease, where higher functioning immune cells would be expected and tumor immune‐suppressive and evasive mechanisms would be less established, and/or in combination with established chemotherapeutic protocols. Alongside, however, it is important to keep in mind that IL‐2's central position at the intersection of adaptive and innate immunity means that strategies manipulating its levels either locally or systemically will directly affect the broader immune landscape. At this time there are few data on how the administration of IL‐2 affects other nontargeted immune subsets, and such findings might prove important for avoiding treatment‐associated side effects or realizing the full efficacy of IL‐2 as a therapeutic agent.

## 4 CONCLUSIONS AND PERSPECTIVES

It is a testament to IL‐2's importance and enduring intrigue that it has been so actively researched for the past 50 years, and we are still making new discoveries even now. Here, we have highlighted the roles of IL‐2 in adaptive and innate immunity, and at their intersection, confirming IL‐2 as a key factor in the maintenance of immune homeostasis across multiple cell types (Fig. 1). Furthermore, many of these findings have now been successfully translated into effective immunotherapies. The overview of currently running clinical studies assures that IL‐2 use in immunotherapy will be further expanding in the future, most probably broadening amount of clinical protocols and well as targeted disorders.

## AUTHORSHIP

KB wrote manuscript and prepared the figures, JF conceptualized and wrote manuscript.

## DISCLOSURES

The authors declare no conflicts of interest.

## References

[1] Morgan DA , Ruscetti FW , Gallo R . Selective in vitro growth of T lymphocytes from normal human bone marrows. Science. 1976;193:1007‐1008. 181845 10.1126/science.181845

[2] Gillis S , Smith KA . Long term culture of tumour‐specific cytotoxic T cells. Nature. 1977;268:154‐156. 145543 10.1038/268154a0

[3] Malek TR , Castro I . Interleukin‐2 receptor signaling: at the interface between tolerance and immunity. Immunity. 2010;33:153‐165. 20732639 10.1016/j.immuni.2010.08.004 PMC2946796

[4] Klatzmann D , Abbas AK . The promise of low‐dose interleukin‐2 therapy for autoimmune and inflammatory diseases. Nat Rev Immunol. 2015;15:283‐294. 25882245 10.1038/nri3823

[5] Durand DB , Shaw JP , Bush MR , Replogle RE , Belagaje R , Crabtree GR . Characterization of antigen receptor response elements within the interleukin‐2 enhancer. Mol Cell Biol. 1988;8:1715‐1724. 3260003 10.1128/mcb.8.4.1715-1724.1988 PMC363332

[6] Shaw JP , Utz PJ , Durand DB , Toole JJ , Emmel EA , Crabtree GR . Identification of a putative regulator of early T cell activation genes. Science. 1988;241:202‐205. 20962265

[7] Okamura H , Aramburu J , Garcia‐Rodriguez C , et al. Concerted dephosphorylation of the transcription factor NFAT1 induces a conformational switch that regulates transcriptional activity. Mol Cell. 2000;6:539‐550. 11030334 10.1016/s1097-2765(00)00053-8

[8] Jain J , McCaffrey PG , Miner Z , et al. The T‐cell transcription factor NFATp is a substrate for calcineurin and interacts with Fos and Jun. Nature. 1993;365:352‐355. 8397339 10.1038/365352a0

[9] Bendickova K , Tidu F , De Zuani M , et al. Calcineurin inhibitors reduce NFAT‐dependent expression of antifungal pentraxin‐3 by human monocytes. J Leukocyte Biol. 2020;107:497‐508. 30934147 10.1002/JLB.4VMA0318-138R PMC7064969

[10] Peng SL , Gerth AJ , Ranger AM , Glimcher LH . NFATc1 and NFATc2 together control both T and B cell activation and differentiation. Immunity. 2001;14:13‐20. 11163226 10.1016/s1074-7613(01)00085-1

[11] Yoshida H , Nishina H , Takimoto H , et al. The transcription factor NF‐ATc1 regulates lymphocyte proliferation and Th2 cytokine production. Immunity. 1998;8:115‐124. 9462517 10.1016/s1074-7613(00)80464-1

[12] Hwang ES , Hong JH , Glimcher LH . IL‐2 production in developing Th1 cells is regulated by heterodimerization of RelA and T‐bet and requires T‐bet serine residue 508. J Exp Med. 2005;202:1289‐1300. 16275766 10.1084/jem.20051044 PMC2213245

[13] Macian F , Lopez‐Rodriguez C , Rao A . Partners in transcription: nFAT and AP‐1. Oncogene. 2001;20:2476‐2489. 11402342 10.1038/sj.onc.1204386

[14] Wu Y , Borde M , Heissmeyer V , et al. FOXP3 controls regulatory T cell function through cooperation with NFAT. Cell. 2006;126:375‐387. 16873067 10.1016/j.cell.2006.05.042

[15] Bettelli E , Dastrange M , Oukka M . Foxp3 interacts with nuclear factor of activated T cells and NF‐kappa B to repress cytokine gene expression and effector functions of T helper cells. Proc Natl Acad Sci USA. 2005;102:5138‐5143. 15790681 10.1073/pnas.0501675102 PMC555574

[16] Schreiber SL , Crabtree GR . The mechanism of action of cyclosporin A and FK506. Immunol Today. 1992;13:136‐142. 1374612 10.1016/0167-5699(92)90111-J

[17] Rush D . The impact of calcineurin inhibitors on graft survival. Transplant Rev (Orlando). 2013;27:93‐95. 23743217 10.1016/j.trre.2013.04.003

[18] Vaeth M , Bauerlein CA , Pusch T , et al. Selective NFAT targeting in T cells ameliorates GvHD while maintaining antitumor activity. Proc Natl Acad Sci USA. 2015;112:1125‐1130. 25583478 10.1073/pnas.1409290112 PMC4313840

[19] Nasu R , Nannya Y , Shinohara A , Ichikawa M , Kurokawa M . Favorable outcomes of tacrolimus compared with cyclosporine A for GVHD prophylaxis in HSCT for standard‐risk hematological diseases. Ann Hematol. 2014;93:1215‐1223. 24590535 10.1007/s00277-014-2027-y

[20] Bendickova K , Tidu F , Fric J . Calcineurin‐NFAT signalling in myeloid leucocytes: new prospects and pitfalls in immunosuppressive therapy. EMBO Mol Med. 2017;9:990‐999. 28606994 10.15252/emmm.201707698 PMC5538425

[21] von Spee‐Mayer C , Siegert E , Abdirama D , et al. Low‐dose interleukin‐2 selectively corrects regulatory T cell defects in patients with systemic lupus erythematosus. Ann Rheum Dis. 2016;75:1407‐1415. 26324847 10.1136/annrheumdis-2015-207776

[22] He J , Zhang R , Shao M , et al. Efficacy and safety of low‐dose IL‐2 in the treatment of systemic lupus erythematosus: a randomised, double‐blind, placebo‐controlled trial. Ann Rheum Dis. 2020;79:141‐149. 31537547 10.1136/annrheumdis-2019-215396 PMC6937406

[23] He J , Zhang X , Wei Y , et al. Low‐dose interleukin‐2 treatment selectively modulates CD4(+) T cell subsets in patients with systemic lupus erythematosus. Nat Med. 2016;22:991‐993. 27500725 10.1038/nm.4148

[24] Humrich JY , Riemekasten G . Low‐dose interleukin‐2 therapy for the treatment of systemic lupus erythematosus. Curr Opin Rheumatol. 2019;31:208‐212. 30562181 10.1097/BOR.0000000000000575

[25] Rosenzwajg M , Lorenzon R , Cacoub P , et al. Immunological and clinical effects of low‐dose interleukin‐2 across 11 autoimmune diseases in a single, open clinical trial. Ann Rheum Dis. 2019;78:209‐217. 30472651 10.1136/annrheumdis-2018-214229

[26] Saadoun D , Rosenzwajg M , Joly F , et al. Regulatory T‐cell responses to low‐dose interleukin‐2 in HCV‐induced vasculitis. N Engl J Med. 2011;365:2067‐2077. 22129253 10.1056/NEJMoa1105143

[27] Todd JA , Evangelou M , Cutler AJ , et al. Regulatory T cell responses in participants with type 1 diabetes after a single dose of interleukin‐2: a non‐randomised, open label, adaptive dose‐finding trial. PLoS Med. 2016;13:e1002139. 27727279 10.1371/journal.pmed.1002139 PMC5058548

[28] Rosenzwajg M , Churlaud G , Mallone R , et al. Low‐dose interleukin‐2 fosters a dose‐dependent regulatory T cell tuned milieu in T1D patients. J Autoimmun. 2015;58:48‐58. 25634360 10.1016/j.jaut.2015.01.001 PMC8153751

[29] Hartemann A , Bensimon G , Payan CA , et al. Low‐dose interleukin 2 in patients with type 1 diabetes: a phase 1/2 randomised, double‐blind, placebo‐controlled trial. Lancet Diabetes Endocrinol. 2013;1:295‐305. 24622415 10.1016/S2213-8587(13)70113-X

[30] Yu A , Snowhite I , Vendrame F , et al. Selective IL‐2 responsiveness of regulatory T cells through multiple intrinsic mechanisms supports the use of low‐dose IL‐2 therapy in type 1 diabetes. Diabetes. 2015;64:2172‐2183. 25576057 10.2337/db14-1322

[31] Castela E , Le Duff F , Butori C , et al. Effects of low‐dose recombinant interleukin 2 to promote T‐regulatory cells in alopecia areata. JAMA Dermatol. 2014;150:748‐751. 24872229 10.1001/jamadermatol.2014.504

[32] Koreth J , Matsuoka K , Kim HT , et al. Interleukin‐2 and regulatory T cells in graft‐versus‐host disease. N Engl J Med. 2011;365:2055‐2066. 22129252 10.1056/NEJMoa1108188 PMC3727432

[33] Kennedy‐Nasser AA , Ku S , Castillo‐Caro P , et al. Ultra low‐dose IL‐2 for GVHD prophylaxis after allogeneic hematopoietic stem cell transplantation mediates expansion of regulatory T cells without diminishing antiviral and antileukemic activity. Clin Cancer Res. 2014;20:2215‐2225. 24573552 10.1158/1078-0432.CCR-13-3205 PMC3989436

[34] Matsuoka K , Koreth J , Kim HT , et al. Low‐dose interleukin‐2 therapy restores regulatory T cell homeostasis in patients with chronic graft‐versus‐host disease. Sci Transl Med. 2013;5:179ra43. 10.1126/scitranslmed.3005265 PMC3686517 23552371

[35] Whangbo JS , Kim HT , Mirkovic N , et al. Dose‐escalated interleukin‐2 therapy for refractory chronic graft‐versus‐host disease in adults and children. Blood Adv. 2019;3:2550‐2561. 31471324 10.1182/bloodadvances.2019000631 PMC6737411

[36] Li Y , Liu X , Wang W , et al. Low‐dose IL‐2 expands CD4(+) regulatory T cells with a suppressive function in vitro via the STAT5‐dependent pathway in patients with chronic kidney diseases. Ren Fail. 2018;40:280‐288. 29619880 10.1080/0886022X.2018.1456462 PMC6014482

[37] Xiao J , Yu K , Li M , Xiong C , Wei Y , Zeng Q . The IL‐2/Anti‐IL‐2 complex attenuates cardiac ischaemia‐reperfusion injury through expansion of regulatory T cells. Cell Physiol Biochem. 2017;44:1810‐1827. 29224017 10.1159/000485818

[38] Zhang R , Xi X , Wang C , et al. Therapeutic effects of recombinant human interleukin 2 as adjunctive immunotherapy against tuberculosis: a systematic review and meta‐analysis. PLoS One. 2018;13:e0201025. 30024982 10.1371/journal.pone.0201025 PMC6053227

[39] Tan Q , Min R , Dai GQ , et al. Clinical and immunological effects of rhIL‐2 therapy in eastern chinese patients with multidrug‐resistant tuberculosis. Sci Rep. 2017;7:17854. 29259310 10.1038/s41598-017-18200-5 PMC5736576

[40] Molloy MJ , Zhang W , Usherwood EJ . Cutting edge: iL‐2 immune complexes as a therapy for persistent virus infection. J Immunol. 2009;182:4512‐4515. 19342623 10.4049/jimmunol.0804175 PMC2682335

[41] Tavel JA , Sereti I , Walker RE , et al. A randomized, double‐blinded, placebo‐controlled trial of intermittent administration of interleukin‐2 and prednisone in subjects infected with human immunodeficiency virus. J Infect Dis. 2003;188:531‐536. 12898439 10.1086/377285

[42] Kovacs JA , Vogel S , Metcalf JA , et al. Interleukin‐2 induced immune effects in human immunodeficiency virus‐infected patients receiving intermittent interleukin‐2 immunotherapy. Eur J Immunol. 2001;31:1351‐1360. 11465092 10.1002/1521-4141(200105)31:5<1351::AID-IMMU1351>3.0.CO;2-9

[43] Atkins MB , Kunkel L , Sznol M , Rosenberg SA . High‐dose recombinant interleukin‐2 therapy in patients with metastatic melanoma: long‐term survival update. Cancer J Sci Am 6 Suppl. 2000;1:S11‐4. 10685652

[44] Davar D , Ding F , Saul M , et al. High‐dose interleukin‐2 (HD IL‐2) for advanced melanoma: a single center experience from the University of Pittsburgh Cancer Institute. J Immunother Cancer. 2017;5:74. 28923120 10.1186/s40425-017-0279-5 PMC5604296

[45] Buchbinder EI , Dutcher JP , Daniels GA , et al. Therapy with high‐dose Interleukin‐2 (HD IL‐2) in metastatic melanoma and renal cell carcinoma following PD1 or PDL1 inhibition. J Immunother Cancer. 2019;7:49. 30777131 10.1186/s40425-019-0522-3 PMC6380045

[46] Ahmadzadeh M , Rosenberg SA . IL‐2 administration increases CD4+ CD25(hi) Foxp3+ regulatory T cells in cancer patients. Blood. 2006;107:2409‐2414. 16304057 10.1182/blood-2005-06-2399 PMC1473973

[47] Lim SH , Newland AC , Kelsey S , et al. Continuous intravenous infusion of high‐dose recombinant interleukin‐2 for acute myeloid leukaemia–a phase II study. Cancer Immunol Immunother. 1992;34:337‐342. 1540980 10.1007/BF01741555 PMC11038385

[48] Foa R , Meloni G , Tosti S , et al. Treatment of acute myeloid leukaemia patients with recombinant interleukin 2: a pilot study. Br J Haematol. 1991;77:491‐496. 2025574 10.1111/j.1365-2141.1991.tb08615.x

[49] Macdonald D , Jiang YZ , Gordon AA , et al. Recombinant interleukin 2 for acute myeloid leukaemia in first complete remission: a pilot study. Leukemia Res. 1990;14:967‐973. 2280612 10.1016/0145-2126(90)90109-m

[50] Miller JS , Soignier Y , Panoskaltsis‐Mortari A , et al. Successful adoptive transfer and in vivo expansion of human haploidentical NK cells in patients with cancer. Blood. 2005;105:3051‐3057. 15632206 10.1182/blood-2004-07-2974

[51] Bachanova V , Cooley S , Defor TE , et al. Clearance of acute myeloid leukemia by haploidentical natural killer cells is improved using IL‐2 diphtheria toxin fusion protein. Blood. 2014;123:3855‐3863. 24719405 10.1182/blood-2013-10-532531 PMC4064329

[52] Burns LJ , Weisdorf DJ , DeFor TE , et al. IL‐2‐based immunotherapy after autologous transplantation for lymphoma and breast cancer induces immune activation and cytokine release: a phase I/II trial. Bone Marrow Transplant. 2003;32:177‐186. 12838283 10.1038/sj.bmt.1704086

[53] Burjanadze M , Condomines M , Reme T , et al. In vitro expansion of gamma delta T cells with anti‐myeloma cell activity by Phosphostim and IL‐2 in patients with multiple myeloma. Br J Haematol. 2007;139:206‐216. 17897296 10.1111/j.1365-2141.2007.06754.x

[54] Bamford RN , Grant AJ , Burton JD , et al. The interleukin (IL) 2 receptor beta chain is shared by IL‐2 and a cytokine, provisionally designated IL‐T, that stimulates T‐cell proliferation and the induction of lymphokine‐activated killer cells. Proc Natl Acad Sci USA. 1994;91:4940‐4944. 8197161 10.1073/pnas.91.11.4940 PMC43905

[55] Wang X , Rickert M , Garcia KC . Structure of the quaternary complex of interleukin‐2 with its alpha, beta, and gammac receptors. Science. 2005;310:1159‐1163. 16293754 10.1126/science.1117893

[56] Boyman O , Sprent J . The role of interleukin‐2 during homeostasis and activation of the immune system. Nat Rev Immunol. 2012;12:180‐190. 22343569 10.1038/nri3156

[57] Rochman Y , Spolski R , Leonard WJ . New insights into the regulation of T cells by gamma(c) family cytokines. Nat Rev Immunol. 2009;9:480‐490. 19543225 10.1038/nri2580 PMC2814538

[58] Deho L , Leoni C , Brodie TM , et al. Two functionally distinct subsets of mast cells discriminated By IL‐2‐independent CD25 activities. J Immunol. 2014;193:2196‐2206. 25063866 10.4049/jimmunol.1400516

[59] Jaleco S , Swainson L , Dardalhon V , Burjanadze M , Kinet S , Taylor N . Homeostasis of naive and memory CD4+ T cells: iL‐2 and IL‐7 differentially regulate the balance between proliferation and Fas‐mediated apoptosis. J Immunol. 2003;171:61‐68. 12816983 10.4049/jimmunol.171.1.61

[60] Cheng G , Yu A , Dee MJ , Malek TR . IL‐2R signaling is essential for functional maturation of regulatory T cells during thymic development. J Immunol. 2013;190:1567‐1575. 23315074 10.4049/jimmunol.1201218 PMC3563871

[61] Weist BM , Kurd N , Boussier J , Chan SW , Robey EA . Thymic regulatory T cell niche size is dictated by limiting IL‐2 from antigen‐bearing dendritic cells and feedback competition. NatImmunol. 2015;16:635‐641. 10.1038/ni.3171 PMC4439282 25939026

[62] Owen DL , Mahmud SA , Vang KB , et al. Identification of cellular sources of IL‐2 needed for regulatory T cell development and homeostasis. J Immunol. 2018;200:3926‐3933. 29728511 10.4049/jimmunol.1800097 PMC5988981

[63] Kalia V , Sarkar S . Regulation of effector and memory CD8 T cell differentiation by IL‐2—A balancing act. Front Immunol. 2018;9:2987. 30619342 10.3389/fimmu.2018.02987 PMC6306427

[64] Fontenot JD , Rasmussen JP , Gavin MA , Rudensky AY . A function for interleukin 2 in Foxp3‐expressing regulatory T cells. Nat Immunol. 2005;6:1142‐1151. 16227984 10.1038/ni1263

[65] Wuest SC , Edwan JH , Martin JF , et al. A role for interleukin‐2 trans‐presentation in dendritic cell‐mediated T cell activation in humans, as revealed by daclizumab therapy. Nat Med. 2011;17:604‐609. 21532597 10.1038/nm.2365 PMC3089658

[66] Suzuki H , Kundig TM , Furlonger C , et al. Deregulated T cell activation and autoimmunity in mice lacking interleukin‐2 receptor beta. Science. 1995;268:1472‐1476. 7770771 10.1126/science.7770771

[67] Sadlack B , Lohler J , Schorle H , et al. Generalized autoimmune disease in interleukin‐2‐deficient mice is triggered by an uncontrolled activation and proliferation of CD4+ T cells. Eur J Immunol. 1995;25:3053‐3059. 7489743 10.1002/eji.1830251111

[68] Willerford DM , Chen J , Ferry JA , Davidson L , Ma A , Alt FW . Interleukin‐2 receptor alpha chain regulates the size and content of the peripheral lymphoid compartment. Immunity. 1995;3:521‐530. 7584142 10.1016/1074-7613(95)90180-9

[69] Macian F . NFAT proteins: key regulators of T‐cell development and function. Nat Rev Immunol. 2005;5:472‐484. 15928679 10.1038/nri1632

[70] Shin DS , Jordan A , Basu S , et al. Regulatory T cells suppress CD4+ T cells through NFAT‐dependent transcriptional mechanisms. EMBO Rep. 2014;15:991‐999. 25074018 10.15252/embr.201338233 PMC4198043

[71] Strober W , Fuss IJ , Blumberg RS . The immunology of mucosal models of inflammation. Annu Rev Immunol. 2002;20:495‐549. 11861611 10.1146/annurev.immunol.20.100301.064816

[72] Tanoue T , Atarashi K , Honda K . Development and maintenance of intestinal regulatory T cells. Nat Rev Immunol. 2016;16:295‐309. 27087661 10.1038/nri.2016.36

[73] Laurence A , Tato CM , Davidson TS , et al. Interleukin‐2 signaling via STAT5 constrains T helper 17 cell generation. Immunity. 2007;26:371‐381. 17363300 10.1016/j.immuni.2007.02.009

[74] Salio M , Gasser O , Gonzalez‐Lopez C , et al. Activation of human mucosal‐associated invariant T cells induces CD40L‐dependent maturation of monocyte‐derived and primary dendritic cells. J Immunol. 2017;199:2631‐2638. 28877992 10.4049/jimmunol.1700615 PMC5632842

[75] Waldmann TA . The shared and contrasting roles of IL2 and IL15 in the life and death of normal and neoplastic lymphocytes: implications for cancer therapy. Cancer Immunol Res. 2015;3:219‐227. 25736261 10.1158/2326-6066.CIR-15-0009 PMC4351780

[76] Zelante T , Fric J , Wong AY , Ricciardi‐Castagnoli P . Interleukin‐2 production by dendritic cells and its immuno‐regulatory functions. Front Immunol. 2012;3:161. 22719740 10.3389/fimmu.2012.00161 PMC3376408

[77] Fric J , Zelante T , Wong AY , Mertes A , Yu HB , Ricciardi‐Castagnoli P . NFAT control of innate immunity. Blood. 2012;120:1380‐1389. 22611159 10.1182/blood-2012-02-404475

[78] Granucci F , Feau S , Angeli V , Trottein F , Ricciardi‐Castagnoli P . Early IL‐2 production by mouse dendritic cells is the result of microbial‐induced priming. J Immunol. 2003;170:5075‐5081. 12734352 10.4049/jimmunol.170.10.5075

[79] Granucci F , Vizzardelli C , Pavelka N , et al. Inducible IL‐2 production by dendritic cells revealed by global gene expression analysis. Nat Immunol. 2001;2:882‐888. 11526406 10.1038/ni0901-882

[80] Slack EC , Robinson MJ , Hernanz‐Falcon P , et al. Syk‐dependent ERK activation regulates IL‐2 and IL‐10 production by DC stimulated with zymosan. Eur J Immunol. 2007;37:1600‐1612. 17458858 10.1002/eji.200636830

[81] Xu S , Huo J , Lee KG , Kurosaki T , Lam KP . Phospholipase Cgamma2 is critical for Dectin‐1‐mediated Ca2+ flux and cytokine production in dendritic cells. J Biol Chem. 2009;284:7038‐7046. 19136564 10.1074/jbc.M806650200 PMC2652331

[82] Herbst S , Shah A , Mazon Moya M , et al. Phagocytosis‐dependent activation of a TLR9‐BTK‐calcineurin‐NFAT pathway co‐ordinates innate immunity to Aspergillus fumigatus. EMBO Mol Med. 2015;7:240‐258. 25637383 10.15252/emmm.201404556 PMC4364943

[83] Strijbis K , Tafesse FG , Fairn GD , et al. Bruton's Tyrosine Kinase (BTK) and Vav1 contribute to Dectin1‐dependent phagocytosis of Candida albicans in macrophages. PLoS Pathogens. 2013;9:e1003446. 23825946 10.1371/journal.ppat.1003446 PMC3694848

[84] Bercusson A , Colley T , Shah A , Warris A , Armstrong‐James D . Ibrutinib blocks Btk‐dependent NF‐kB and NFAT responses in human macrophages during Aspergillus fumigatus phagocytosis. Blood. 2018;132:1985‐1988. 30021784 10.1182/blood-2017-12-823393 PMC6450054

[85] Goodridge HS , Simmons RM , Underhill DM . Dectin‐1 stimulation by Candida albicans yeast or zymosan triggers NFAT activation in macrophages and dendritic cells. J Immunol. 2007;178:3107‐3115. 17312158 10.4049/jimmunol.178.5.3107

[86] Zanoni I , Ostuni R , Capuano G , et al. CD14 regulates the dendritic cell life cycle after LPS exposure through NFAT activation. Nature. 2009;460:264‐268. 19525933 10.1038/nature08118

[87] Yu HB , Yurieva M , Balachander A , et al. NFATc2 mediates epigenetic modification of dendritic cell cytokine and chemokine responses to dectin‐1 stimulation. Nucleic Acids Rese. 2015;43:836‐847. 10.1093/nar/gku1369 PMC4333412 25550437

[88] Zelante T , Wong AY , Ping TJ , et al. CD103(+) Dendritic Cells Control Th17 Cell Function in the Lung. Cell Rep. 2015;12:1789‐1801. 26365185 10.1016/j.celrep.2015.08.030

[89] Seyedmousavi S , Davis MJ . Defective calcineurin/NFAT signaling in myeloid cells and susceptibility to aspergillosis in post‐transplant patients. Virulence. 2017;8:1498‐1501. 28922070 10.1080/21505594.2017.1380143 PMC5810483

[90] Mencarelli A , Khameneh HJ , Fric J , et al. Calcineurin‐mediated IL‐2 production by CD11c(high)MHCII(+) myeloid cells is crucial for intestinal immune homeostasis. Nat Commun. 2018;9:1102. 29549257 10.1038/s41467-018-03495-3 PMC5856784

[91] Han D , Walsh MC , Cejas PJ , et al. Dendritic cell expression of the signaling molecule TRAF6 is critical for gut microbiota‐dependent immune tolerance. Immunity. 2013;38:1211‐1222. 23791643 10.1016/j.immuni.2013.05.012 PMC3715143

[92] Zhou L , Chu C , Teng F , et al. Innate lymphoid cells support regulatory T cells in the intestine through interleukin‐2. Nature. 2019;568:405‐409. 30944470 10.1038/s41586-019-1082-x PMC6481643

[93] Decot V , Voillard L , Latger‐Cannard V , et al. Natural‐killer cell amplification for adoptive leukemia relapse immunotherapy: comparison of three cytokines, IL‐2, IL‐15, or IL‐7 and impact on NKG2D, KIR2DL1, and KIR2DL2 expression. Exp Hematol. 2010;38:351‐362. 20172016 10.1016/j.exphem.2010.02.006

[94] Reichlin A , Yokoyama WM . Natural killer cell proliferation induced by anti‐NK1.1 and IL‐2. Immunol Cell Biol. 1998;76:143‐152. 9619484 10.1046/j.1440-1711.1998.00726.x

[95] Aramburu J , Azzoni L , Rao A , Perussia B . Activation and expression of the nuclear factors of activated T cells, NFATp and NFATc, in human natural killer cells: regulation upon CD16 ligand binding. J Exp Med. 1995;182:801‐810. 7650486 10.1084/jem.182.3.801 PMC2192167

[96] Fehniger TA , Cooper MA , Nuovo GJ , et al. CD56bright natural killer cells are present in human lymph nodes and are activated by T cell‐derived IL‐2: a potential new link between adaptive and innate immunity. Blood. 2003;101:3052‐3057. 12480696 10.1182/blood-2002-09-2876

[97] Granucci F , Zanoni I , Pavelka N , et al. A contribution of mouse dendritic cell‐derived IL‐2 for NK cell activation. J Exp Med. 2004;200:287‐295. 15289500 10.1084/jem.20040370 PMC2211974

[98] Santus W , Barresi S , Mingozzi F , et al. Skin infections are eliminated by cooperation of the fibrinolytic and innate immune systems. Sci Immunol. 2017;2. 10.1126/sciimmunol.aan2725 PMC5682945 28939652

[99] Gasteiger G , Hemmers S , Bos PD , Sun JC , Rudensky AY . IL‐2‐dependent adaptive control of NK cell homeostasis. J Exp Med. 2013;210:1179‐1187. 23650439 10.1084/jem.20122571 PMC3674698

[100] Parrish‐Novak J , Dillon SR , Nelson A , et al. Interleukin 21 and its receptor are involved in NK cell expansion and regulation of lymphocyte function. Nature. 2000;408:57‐63. 11081504 10.1038/35040504

[101] Waldmann TA . The biology of interleukin‐2 and interleukin‐15: implications for cancer therapy and vaccine design. Nat Rev Immunol. 2006;6:595‐601. 16868550 10.1038/nri1901

[102] Rosenberg SA . IL‐2: the first effective immunotherapy for human cancer. J Immunol. 2014;192:5451‐5458. 24907378 10.4049/jimmunol.1490019 PMC6293462

[103] Dimberg J , Shamoun L , Landerholm K , Andersson RE , Kolodziej B , Wagsater D . Genetic variants of the IL2 gene related to risk and survival in patients with colorectal cancer. Anticancer Res. 2019;39:4933‐4940. 31519598 10.21873/anticanres.13681

[104] Dhupkar P , Gordon N . Interleukin‐2: old and new approaches to enhance immune‐therapeutic efficacy. Adv Exp Med Biol. 2017;995:33‐51. 28321811 10.1007/978-3-319-53156-4_2

[105] Mitra S , Ring AM , Amarnath S , et al. Interleukin‐2 activity can be fine tuned with engineered receptor signaling clamps. Immunity. 2015;42:826‐838. 25992859 10.1016/j.immuni.2015.04.018 PMC4560365

[106] Lutz MB , Baur AS , Schuler‐Thurner B , Schuler G . Immunogenic and tolerogenic effects of the chimeric IL‐2‐diphtheria toxin cytocidal agent Ontak((R)) on CD25(+) cells. Oncoimmunology. 2014;3:e28223. 25050193 10.4161/onci.28223 PMC4091105

[107] Ohmachi K , Ando K , Ogura M , et al. E7777 in Japanese patients with relapsed/refractory peripheral and cutaneous T‐cell lymphoma: a phase I study. Cancer Sci. 2018;109:794‐802. 29363235 10.1111/cas.13513 PMC5834772

[108] Rafei‐Shamsabadi D , Lehr S , von Bubnoff D , Meiss F . Successful combination therapy of systemic checkpoint inhibitors and intralesional interleukin‐2 in patients with metastatic melanoma with primary therapeutic resistance to checkpoint inhibitors alone. Cancer Immunol Immunother. 2019;68:1417‐1428. 31422446 10.1007/s00262-019-02377-x PMC11028275

[109] Boyman O , Krieg C , Letourneau S , Webster K , Surh CD , Sprent J . Selectively expanding subsets of T cells in mice by injection of interleukin‐2/antibody complexes: implications for transplantation tolerance. Transplant Proc. 2012;44:1032‐1034. 22564618 10.1016/j.transproceed.2012.01.093

[110] Sun Z , Ren Z , Yang K , et al. A next‐generation tumor‐targeting IL‐2 preferentially promotes tumor‐infiltrating CD8(+) T‐cell response and effective tumor control. Nat Commun. 2019;10:3874. 31462678 10.1038/s41467-019-11782-w PMC6713724

[111] Muul LM , Spiess PJ , Director EP , Rosenberg SA . Identification of specific cytolytic immune responses against autologous tumor in humans bearing malignant melanoma. J Immunol. 1987;138:989‐995. 3100623

[112] Krasnova Y , Putz EM , Smyth MJ , Souza‐Fonseca‐Guimaraes F . Bench to bedside: nK cells and control of metastasis. Clinical Immunol. 2017;177:50‐59. 26476139 10.1016/j.clim.2015.10.001

[113] Souza‐Fonseca‐Guimaraes F , Cursons J , Huntington ND . The emergence of natural killer cells as a major target in cancer immunotherapy. Trends Immunol. 2019;40:142‐158. 30639050 10.1016/j.it.2018.12.003

[114] Parkhurst MR , Riley JP , Dudley ME , Rosenberg SA . Adoptive transfer of autologous natural killer cells leads to high levels of circulating natural killer cells but does not mediate tumor regression. Clin Cancer Res. 2011;17:6287‐6297. 21844012 10.1158/1078-0432.CCR-11-1347 PMC3186830

[115] Ni J , Miller M , Stojanovic A , Garbi N , Cerwenka A . Sustained effector function of IL‐12/15/18‐preactivated NK cells against established tumors. J Exp Med. 2012;209:2351‐2365. 23209317 10.1084/jem.20120944 PMC3526364

[116] Geller MA , Miller JS . Use of allogeneic NK cells for cancer immunotherapy. Immunotherapy. 2011;3:1445‐1459. 22091681 10.2217/imt.11.131 PMC3292871

[117] Hallett WHD , Ames E , Alvarez M , et al. Combination therapy using IL‐2 and anti‐CD25 results in augmented natural killer cell‐mediated antitumor responses. Biol Blood Marrow Transplant. 2008;14:1088‐1099. 18804038 10.1016/j.bbmt.2008.08.001 PMC2735407

[118] Zhang J , Zheng H , Diao Y . Natural killer cells and current applications of chimeric antigen receptor‐modified NK‐92 cells in tumor immunotherapy. Int J Mol Sci. 2019;20. 10.3390/ijms20020317 PMC6358726 30646574

[119] Chrobok M , Dahlberg CIM , Sayitoglu EC , et al. Functional assessment for clinical use of serum‐free adapted NK‐92 cells. Cancers. 2019;11. 10.3390/cancers11010069 PMC6356567 30634595

[120] Williams BA , Wang XH , Leyton JV , et al. CD16(+)NK‐92 and anti‐CD123 monoclonal antibody prolongs survival in primary human acute myeloid leukemia xenografted mice. Haematologica. 2018;103:1720‐1729. 29976748 10.3324/haematol.2017.187385 PMC6165813

[121] Lowdell MW , Lamb L , Hoyle C , Velardi A , Prentice HG . Non‐MHC‐restricted cytotoxic cells: their roles in the control and treatment of leukaemias. Br J Haematol. 2001;114:11‐24. 11472339 10.1046/j.1365-2141.2001.02906.x

[122] Rubnitz JE , Inaba H , Ribeiro RC , et al. NKAML: a pilot study to determine the safety and feasibility of haploidentical natural killer cell transplantation in childhood acute myeloid leukemia. J Clin Oncol. 2010;28:955‐959. 20085940 10.1200/JCO.2009.24.4590 PMC2834435

[123] Kunzmann V , Bauer E , Feurle J , Weissinger F , Tony HP , Wilhelm M . Stimulation of gammadelta T cells by aminobisphosphonates and induction of antiplasma cell activity in multiple myeloma. Blood. 2000;96:384‐392. 10887096

[124] Mariani S , Muraro M , Pantaleoni F , et al. Effector gammadelta T cells and tumor cells as immune targets of zoledronic acid in multiple myeloma. Leukemia. 2005;19:664‐670. 15744346 10.1038/sj.leu.2403693

[125] Viey E , Fromont G , Escudier B , et al. Phosphostim‐activated gamma delta T cells kill autologous metastatic renal cell carcinoma. J Immunol. 2005;174:1338‐1347. 15661891 10.4049/jimmunol.174.3.1338

[126] Wilhelm M , Smetak M , Schaefer‐Eckart K , et al. Successful adoptive transfer and in vivo expansion of haploidentical gammadelta T cells. J Transl Med. 2014;12:45. 24528541 10.1186/1479-5876-12-45 PMC3926263

